# Effects of Self-Compassion Training on Work-Related Well-Being: A Systematic Review

**DOI:** 10.3389/fpsyg.2021.630798

**Published:** 2021-04-23

**Authors:** Yasuhiro Kotera, William Van Gordon

**Affiliations:** Human Sciences Research Centre, University of Derby, Derby, United Kingdom

**Keywords:** self-compassion, work-related well-being, systematic review, work mental health, workers, workplace mental health, caring professions, self-care

## Abstract

Self-compassion, sharing some commonalities with positive psychology 2.0 approaches, is associated with better mental health outcomes in diverse populations, including workers. Due to the COVID-19 pandemic, there is heightened awareness of the importance of self-care for fostering mental health at work. However, evidence regarding the applications of self-compassion interventions in work-related contexts has not been systematically reviewed to date. Therefore, this systematic review aimed to synthesize and evaluate the utility of self-compassion interventions targeting work-related well-being, as well as assess the methodological quality of relevant studies. Eligible articles were identified from research databases including ProQuest, PsycINFO, Science Direct, and Google Scholar. The quality of non-randomized trials and randomized controlled trials was assessed using the Newcastle-Ottawa Scale and the Quality Assessment Table, respectively. The literature search yielded 3,387 titles from which ten studies met the inclusion criteria. All ten studies reported promising effects of self-compassion training for work-related well-being. The methodological quality of these studies was medium. All ten studies recruited workers in a caring field and were mostly conducted in Western countries. The Self-Compassion Scale or its short-form was used in almost all instances. Findings indicate that self-compassion training can improve self-compassion and other work-related well-being outcomes in working populations. However, in general, there is need for greater methodological quality in work-related self-compassion intervention studies to advance understanding regarding the applications and limitations of this technique in work contexts. Furthermore, future studies should focus on a broader range of employee groups, including non-caring professions as well as individuals working in non-Western countries.

## Introduction

In the United Kingdom, workplace mental health problems resulted in 16 million lost working days in 2016 (Office for National Statistics, 2017), costing £40 billion and £25 billion to employers and the government, respectively (Stevenson and Farmer, 2017). In other European countries such as the Netherlands, in 2014 the cost of poor work mental health amounted to 3.3% of GDP (€746 billion), mainly as a result of lost employment and reduced work productivity (Organization for Economic Cooperation and Development, 2014). In Asian countries such as Japan, 60% of workers experience intense anxiety and stress (Ministry of Health Labour Welfare, 2010), and the number of worker compensation claims for mental health problems increased from 200 in 2000 to 1,800 in 2018 (Ministry of Health Labour Welfare, 2019).

Awareness of such workplace mental health problems has become further heightened due to the coronavirus disease 2019 (COVID-19) pandemic. For example, a recent literature review identified that workplace stress and depression were associated with the pandemic due to threat and worry about contagion, infobesity (i.e., pandemic related information overload), quarantine and reduction of social contact, stigma, and job insecurity (Hamouche, 2020). Conversely, organizational COVID-19 mental health preventative measures are positively associated with job performance and psychological well-being (Sasaki et al., 2020).

Growing awareness of the negative impact of workplace mental health problems has resulted in increased interest into psychotherapeutic interventions that can help to improve mental health and well-being at work (Sustainable Development Goals; United Nations., 2015). One such approach that has emerged as a topic of empirical interest are self-compassion interventions (Barnard and Curry, 2011; López et al., 2015; Kotera et al., 2019c). In work contexts, self-compassion is associated with improvements in (for example) healthy self-care behaviors (Horan and Taylor, 2018), optimism and organizational integrity (Simões et al., 2016), perceived organizational compassion and threat (Henshall et al., 2018), compassion for others (Mills et al., 2017), job satisfaction (Abaci and Arda, 2013), and burnout and barriers to compassion (Dev et al., 2018). Furthermore, interventions specifically targeting self-compassion have led to salutary workplace outcomes such as lower stress (Mahon et al., 2017), reduced burnout (Eriksson et al., 2018), and enhanced resilience (Delaney, 2018).

Self-compassion is described by Neff (2003a) as having a keen awareness of suffering in oneself and others, which comprises three components: (i) self-kindness (being kind and understanding towards oneself), (ii) common humanity (knowing that suffering is part of human life), and (iii) mindfulness (being present here and now). Self-compassion is related to, but distinct from, compassion, which refers more to being sensitive to suffering in others, while strongly wishing to relieve that suffering (Goetz et al., 2010; Shonin et al., 2017). Nevertheless, self-compassion is understood to play an important role in cultivating compassion for others, because self-compassion views failure and suffering as part of human experience, which allows others' failure and suffering to be recognized as “shared human fallibility” (Neff, 2003a, p. 87).

Self-compassion is understood to facilitate the process of being open to one's personal failures, inadequacies, and suffering (Neff, 2003a). However, unlike self-esteem which is often tied to feeling valued by others (Leary and MacDonald, 2003), self-compassion is more concerned with caring for oneself. This self-care competency includes open-hearted awareness of all aspects of oneself, leading to more resilience and stability during difficult times (Neff and Vonk, 2009). This is consistent with studies showing that self-compassion can nurture forgiveness and kindness toward self, as well as reduce guilt, shame, and rumination derived from mistakes (Adams and Leary, 2007; Mantzios et al., 2015).

Self-compassion is sometimes grouped together with other Buddhist-derived practices, such as mindfulness, loving-kindness, compassion, and non-attachment. However, the extent to which self-compassion reflects an entirely Buddhist-derived construct is currently unclear (Shonin et al., 2014). For example, the self-kindness component of self-compassion primarily refers to happiness for oneself, whereas Buddhism emphasizes happiness for others, particularly in the context of the core Buddhist teaching on the four immeasurable attitudes of (i) loving-kindness, (ii) compassion, (iii) appreciative joy, and (iv) equanimity for all beings (Peng and Shen, 2012; Shonin et al., 2015). Consequently, it could be argued that the self-emphasis aspect of self-compassion runs antithetical to Buddhism's focus on others and on dismantling self-oriented schemas (e.g., no-self realization; Wang, 2020). Nevertheless, self-compassion clearly shares some similarities with certain Buddhist practices, particularly mindfulness, in which there is a common focus on present moment awareness and self-regulation of thoughts and emotions (Neff, 2003a; Bishop et al., 2004). Indeed, psychometric scales for self-compassion and mindfulness demonstrate good levels of positive association (Birnie et al., 2010; Hollis-Walker and Colosimo, 2011).

In addition to sharing some properties with mindfulness, studies based on a work-related context have shown that self-compassion is a correlate and predictor of a range of positive mental health outcomes (e.g., Montero-Marin et al., 2018; Kotera et al., 2019b, 2020). For example, self-compassionate workers tend to have higher levels of well-being and experience less burnout and emotional exhaustion (Alkema et al., 2008; Dev et al., 2018). Self-compassion is also negatively associated with anxious and avoidant attachments, and positively associated with work performance, organizational prosocial behaviors and employment retention (Reizer, 2019).

Such associations may be explained by Gilbert's (2010) affect regulatory model whereby self-compassion activates an individual's soothing system (related to feeling safe, connected and cared for), as opposed to drive (related to excitement, striving and achieving) and threat (related to fear, anxiety and anger) systems that are more associated with mental distress. Thus, self-compassion is understood to be a good predictor of well-being at work because work-related well-being outcomes are impacted by self-kindness as part of adaptive soothing system functioning (Pauley and McPherson, 2010).

These characteristics of self-compassion appear to be aligned with the second wave of positive psychology (PP 2.0), a new form of positive psychology that aims to attain the best in individuals, organizations and society, by communicating with the dark side of human existence through an understanding of ying-yang dialectical principles, rather than a liner interpretation of happiness as characterized by traditional first wave positive psychology approaches (Wong, 2011a). All emotions, including negative ones, can have positive meaning in respect of our well-being (Vanderheiden and Mayer, 2017). As the Chinese/Japanese character for “happiness (幸)” is built upon “hardship (辛)”, PP 2.0 does not position the dark side of human existence as a problem, rather it recognizes it as a necessity (Wong, 2020a), relating to meaningfulness (Wong, 2011b, 2012). Moreover, PP 2.0 focuses on both individual well-being and the larger picture of humanity, instead of solely focusing on individual happiness and success (Wong, 2017a). PP 2.0 entails a sense of acceptance, harmony and feeling at home in one's quest for true well-being (Wong, 2017b), which can be even more crucial to counter mental health difficulties associated with COVID-19 (Wong, 2020b). These perspectives also appear to be integral to self-compassion, where hardship as part of natural human life (common humanity) is accepted and experienced non-judgmentally, instead of problematizing and reacting to it (Neff, 2003a).

Notwithstanding the aforementioned challenges for mental health in the workplace as well as the promising findings for self-compassion as a remedial approach, a systematic review specifically focusing on the evidence for self-compassion interventions in a workplace context has not been undertaken to date. Therefore, the purpose of this paper was to conduct a systematic review of self-compassion interventions focusing on outcomes relating to improving workplace well-being.

## Methods

### Design

A systematic review was conducted based on the Preferred Reporting Items for Systematic Reviews and Meta-Analyses (PRISMA; Moher et al., 2009). In addition to a rigorous search and selection process, study quality, means of assessment and results were all considered in order to support the validity of the systematic review (Klassen et al., 1998). The extended version of the PICO (population, intervention, control, and outcomes) format (Boland et al., 2013) was used to establish the research question. The CIMO (Context, Intervention, Mechanism, and Outcome; Denyer and Tranfield, 2009) is another well-used format in organizational contexts, however the extended PICO was preferred because self-compassion originated in clinical practice. The main research questions for this systematic review were (i) how effective is self-compassion for improving outcomes relevant to work-related well-being? and (ii) what quantity and quality of evidence is there? A decision was made not to conduct a meta-analysis as a preliminary literature search indicated there was an insufficient number of randomized controlled trials meeting the eligibility criteria (Janssen et al., 2018).

### Literature Search

After consulting a subject librarian (Rojon et al., 2011), the following electronic research databases were used to conduct a comprehensive literature search: ProQuest, PsycINFO, Science Direct, and Google Scholar via EBSCO. The literature search focusing on where, when, who, how, what, and why (Callahan, 2010). Only papers published before 31st December 2019 were considered eligible for inclusion in this systematic review. Among the papers retrieved with search terms “self-compassion” or “self compassion” (*n* = 3,387), papers that had “work^*^,” “occupation^*^,” “profession^*^,” “staff,” “job,” “employee^*^,” “management,” “business,” and “organization^*^” in the title or abstract were shortlisted for further examination (*n* = 203). The selection of such terms was informed by prior systematic reviews evaluating psychological interventions in the workplace (e.g., Ravalier et al., 2016; Kotera et al., 2019a). The first author completed the literature search, which was then reviewed by the second author. Manual reference searches on previous systematic reviews of self-compassion interventions were also undertaken (Rojon et al., 2011).

### Selection of Studies and Outcomes

The inclusion criteria for further analysis were that the paper needed to (i) be published in a peer-reviewed academic journal written in English language, (ii) report an empirical intervention study (i.e., pre- and post-intervention scores of dependent variables) of a self-compassion intervention, and (iii) involve full-time or part-time workers as participants. Papers were excluded if they (i) did not evaluate an intervention (e.g., papers that only introduced and/or discussed concepts or research protocols), (ii) employed a single-participant design (i.e., case studies), and/or (iii) did not assess work-related well-being outcomes (see Table 1).

**Table 1:** Extended PICO for this systematic review.

**Review questions**	**(i) How effective is self-compassion for work-related well-being outcomes? and (ii) What quantity and quality of evidence is there?**	
	**Inclusion criteria**	**Exclusion criteria**
Population	Workers in an organization (i.e., employees > 18 years old)	< 18 years and non-work samples
Intervention	Interventions that focused on self-compassion^*^	Other interventions
Comparator	Any comparator including non-intervention	
Outcomes	Work-related well-being outcomes^**^	Other outcomes
Study design	Empirical intervention studies	Single participant case studies, cross-sectional studies, qualitative studies, reviews, discussion articles, articles introducing theories/concepts/ models/applications
Other	Published in a peer-reviewed journal in English language	

* *training that did not focus on self-compassion was excluded (e.g., resilience training that increased self-compassion; Kinman and Grant, 2017), however studies integrating mindfulness or other techniques as part of self-compassion training were included (e.g., the Mindful Self-Compassion Program; Germer and Neff, 2019).*

** *engagement, stress, distress, well-being, security, safety, satisfaction, burnout, resilience, efficacy, caring, trust, mindfulness, creativity, hope, emotional intelligence (determined by reviewing key organizational psychology journals in the last 5 years)*.

### Outcome Measures

Outcomes relevant to work-related well-being were selected by reviewing papers published in organizational psychology journals (i.e., included in the Scimago Journal and Country Rank such as *Annual Review of Organizational Psychology, Organizational Behavior*, and *Human Resource Development Quarterly*) during the last 5 years (i.e., to ensure that outcomes corresponded to current foci in organizational psychology research and practice; Kotera et al., 2019a). Eligible outcomes were deemed to be stress, distress, well-being, work-engagement, security, work safety, job satisfaction, burnout, resilience, efficacy, caring, trust, mindfulness, creativity, hope, and emotional intelligence.

### Data Extraction and Synthesis

The lead author comprehensively reviewed all the search results, and if the title of the article implied a fit with the eligibility criteria, the articles were shortlisted for possible inclusion (*n* = 39). The second author reviewed the selection process to minimize any potential bias. Once the second author had reviewed the selection process, full-texts of the shortlisted articles were examined by both authors independently, who then met to discuss which studies met the eligibility criteria. Forward and backward reference searches of relevant articles identified no additional eligible studies.

An extended format for data extraction, designed by Sturt et al. (2012), was used to present key information of included studies, covering: publication details (authors, year, and country), study design and setting, participant characteristics, details of demographic data, intervention details, outcome measures, and study findings (see Table 2).

**Table 2:** Study details of selected papers exploring the effects of self-compassion training on work-related well-being outcomes.

**Author(s), year. Country**.	**Sample and setting**	**Intervention details**	**Measures**	**Findings**
Maratos et al. (2019). United Kingdom.	20 to 18 members of staff within a school specializing in educating 11–18-year-old children/adolescents excluded from mainstream education (2 did not complete the FSCRS and SCS-SF thus *n* = 18, and 1 did not complete MBI at post-assessment *n* = 19). Within-subject pre-post.	Six 2.5-h sessions on compassionate mind training (CMT) program over 12 weeks (one school term).	Maslach Burnout Inventory (MBI), Depression Anxiety and Stress Scale 21 (DASS), Forms of Self-Criticism and Self-Reassuring Scale (FSCRS), Self-Compassion Scale Short-Form (SCS-SF), CMT Practice Scale, administered 2-month and 1-week before, and 1-month after intervention.	Self-compassion and reassured-self increased from pre-intervention to post-intervention with large effects.
Sansó et al. (2019). Spain.	50 professional carers, divided into Mindful-Based Stress Reduction Training (MBSRT) and Compassion Cultivation Training (CCT), 25 each (17 females and 2 males with age 42.16 ± 7.67 in MBSRT and 14 females and 5 males with age 48.95 ± 9.84 in CCT). Self-selected non-equivalent groups design.	Each training was provided for 60 h; extensive weekend training × 3 (3-month interval each time) and weekly sessions.	Five-Facets Mindfulness Questionnaire (FFMQ), Interpersonal Reactivity Index (IRI), SCS, Short version of the Professional Quality of Life Scale (Short ProQol), administered pre-post.	CCT increased non-reactivity and perspective taking significantly, while reduced self-judgement significantly.
Smith et al. (2019). United Kingdom.	Multi-disciplinary team (*n* = 34 at pre-intervention and *n* = 27 at post-intervention) in adult eating disorder wards. Age and gender balance not reported. Fifty-five patient participants were excluded, as non-workers. Within-subject pre-post.	8 workshops focusing on well-being including self-compassion, positive communication, and stress-coping.	Five-item well-being questionnaire.	Self-worth to take time for themselves, and feeling good on the day increased significantly from pre- to post-intervention.
Delaney (2018). United Kingdom.	13 female nurses specialized in Cancer Care, Cardiology, Maternity, Midwifery, Intensive Care, and Urology. Average age 44 years old, and average work experience 21 years old (representable for general nurses).	Mindful Self-Compassion training (MSC): 8 of 2.5-h weekly sessions and a half-day retreat. Daily practice was encouraged.	SCS, Frieburg Short Mindfulness Scale, ProQOL, and Conor-Davidson Resilience Scale	Self-compassion and resilience increased significantly, and secondary trauma and burnout decreased significantly (all large effect size).
Rao and Kemper (2017). United States.	153 health professionals including nurses, physicians, and social workers, attended a compassion session, out of 177 (148 females, 29 males) who attended at least one of the three sessions. Within-subject pre-post.	1-h online meditation sessions focusing on compassion. The other 2 sessions focused on gratitude and positive words were excluded from this review.	SCS-SF and Confidence in Providing Compassionate Care Scale	Significant improvements in self-compassion overall, and within each subcategory of self-compassion: self-kindness, self-judgment, common humanity, isolation, mindfulness, and overidentification. Also increased confidence in providing compassionate care to others.
Sansó et al. (2017). Spain.	19 professional caregivers of patients with intellectual disability (18 females, mean age = 40.47 years old). Within-subject pre-post.	Cultivating Emotional Balance program, based on mindfulness and compassion. 42 h in total (4-h sessions × 10 and 2-h session × 1).	SCS, FFMQ, EQ, Professional Self-Care Scale (PSCS), Brief Symptom Questionnaire (BSQ)	Meaningful increases in both the FFM and de-centering. Improved self-care and self-compassion. Reduced depression, anxiety, panic, and somatized illness.
Scarlet et al. (2017). United States.	62 (12 males and 50 females; Age 51.23 ± 10.77 years old). Within-subject pre-post.	Compassion cultivation training (8 times of 2-h weekly group sessions).	SCS-SF, Fears of Compassion Scale, Toronto Mindfulness Scale, Copenhagen Burnout Inventory (CBI), Brief Index of Affective Job Satisfaction, and Interpersonal Conflict Scale, administered in the first, middle, and last weeks of CCT, with 1-month follow-up.	Significant improvements in participants' self-compassion, mindfulness, and interpersonal conflict scores
Suyi et al. (2017). Singapore.	37 mental health professionals (7 males and 30 females, 19 of them in age range 25-35 years old). Within-subject pre-post.	2-h weekly mindfulness session for 6 weeks, including engagement with practice, awareness on stuckness, reacting and responding to stress, communication, and compassion toward others and self. Additional discussion time among participants and 30-min daily meditation homework.	SCS-SF, FFMQ, CS, PSS, and Oldenburg Burnout Inventory (OBI), measured pre-post, and a 3-month follow-up.	Significant improvement in 4 of the 5 mindfulness facets (observe, describe, non-judge, and non-react) and self-compassion both from pre to post, and pre to follow-up. Significant improvement in compassion for others but only significant between pre and post (not pre and follow-up). Significant reduction in stress but only between pre and post, not pre and follow-up.
Beshai et al. (2016). United Kingdom.	89 teachers and staff from secondary school (62 females and 27 males), self-selected to either intervention group (*n* = 49, who received school-based mindfulness training) or comparison group (*n* = 40, who received general mindfulness training). Nonequivalent groups design.	8-week school-based mindfulness training, consisting of 9 sessions (75 min each), covering body scan, cultivating self-compassion and discussion on school-related issues, with homework: 10–40 min for 6 days a week. Comparison group received a group intervention based on MBSR and MBCT.	SCS (only 2 subscales: self-judgement and self-kindness), FFMQ, PSS, Warwick-Edinburgh Mental Well-being Scale (WEMWBS), administered pre- and post-training.	Stress decreased and well-being increased in the intervention group, in comparison to the comparison group. Large effect on all outcome measures in the intervention group, when controlling for baseline differences with comparison group. Self-compassion increased from pre-intervention to post-intervention in intervention group while it did not in comparison group.
Pidgeon et al. (2014). Australia.	44 human services professionals (40 females and 4 males, Age 40.7 ± 12.28 years) divided into the intervention group and control group. RCT.	Brief Mindfulness with Metta Training Program (MMTP), targeting the enhancement of mindfulness and self-compassion in a retreat format	Resilience Scale, FFMQ and SCS administered pre-/post-training, 1 and 4 months follow-up.	Significant improvements observed in mindfulness and self-compassion at 1 and 4 months post-intervention, and in resilience at 4 months post-intervention for the intervention group.

### Quality Scoring: Assessing the Risk of Bias

The Newcastle-Ottawa Scale (NOS) was used to appraise the methodological quality and risk of bias for non-randomized studies (Wells et al., 2000). Both authors conducted the quality scoring for each study, using star marks, from 0 to 9 (high risk: 0-3, medium risk: 4-6, low risk: 7-9) in respect of: (i) representativeness of study group selection (four stars maximum), (ii) comparability of groups (two stars maximum), and (iii) ascertainment of either the exposure or outcome of interest (three stars maximum). Because the NOS originated in medical research, some adjustments were made in this review focusing on work-related well-being. More specifically, the word “exposure” was changed to “intervention” (e.g., “Ascertainment of intervention”). Similarly, the fourth scale item (in the “Selection” category) was changed from “Demonstration that outcome of interest was not present at start of study” to “Demonstration that the measured outcome was assessed before the intervention.” Furthermore, instead of medical records, a star was marked if the outcome was measured using a validated psychometric scale in the first item of “Outcome” (i.e., “Assessment of Outcome”).

The Quality Assessment Table of Randomized Controlled Trials (Brown et al., 2013) was used to assess the methodological quality of randomized controlled trials. To be compatible with the NOS rating, rating was completed using a star (^*^) for “yes,” blank for “no,” and “NA” for “not applicable,” as the primary interest was whether each research item was adequately reported. These assessments were performed by both of authors independently, who discussed any disagreement. The star rating ranged from 0 to 14; high risk: 0-4, medium risk: 5-9, low risk: 10-14.

## Results

### Search Results

**Figure 1:**
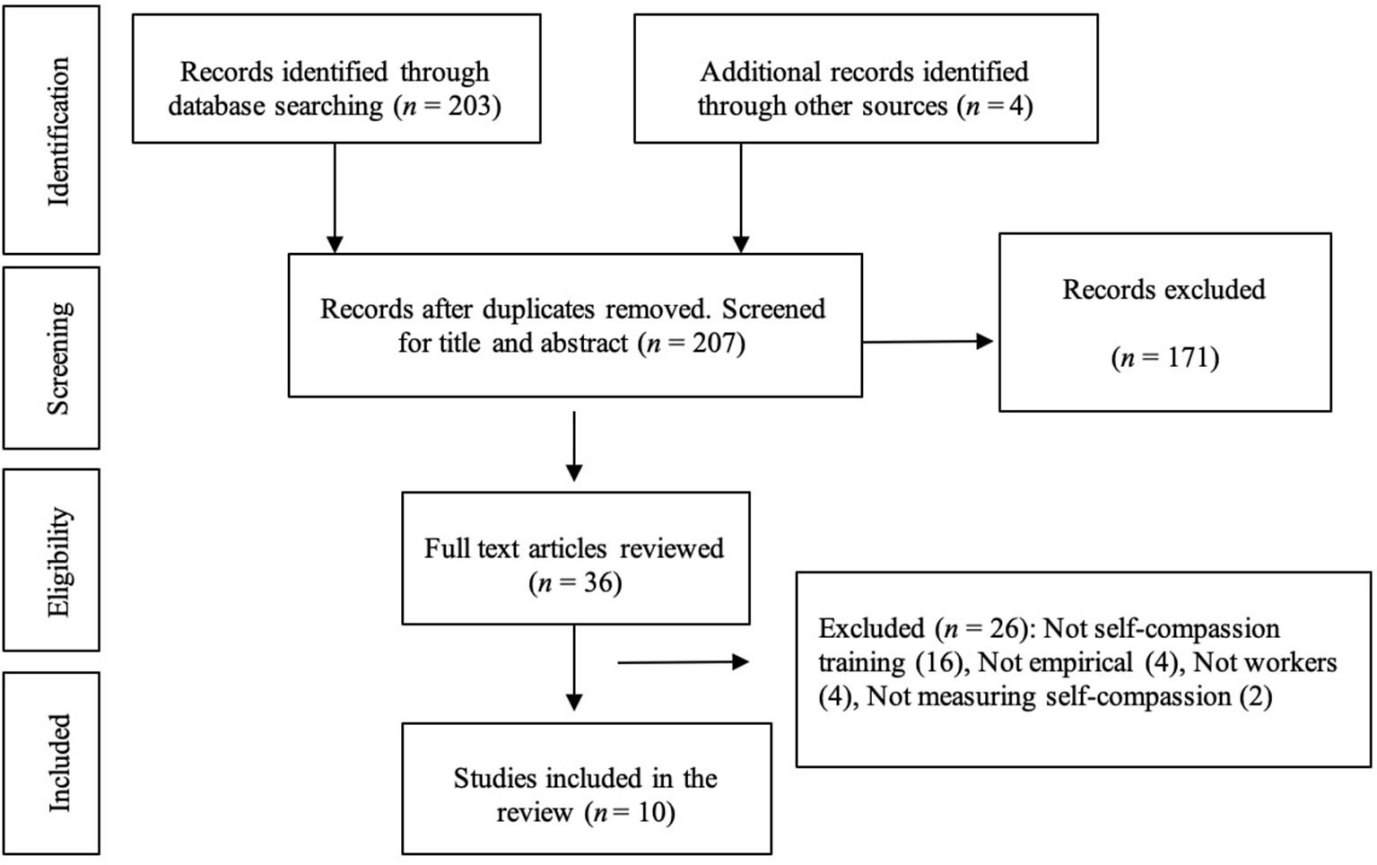
PRISMA flow diagram of the article selection process.

The initial comprehensive literature search identified a total of 207 papers, including four papers (Shapiro et al., 2005; Frank et al., 2013; Roeser et al., 2013; Beshai et al., 2016) identified via manual searches of previous self-compassion reviews (Raab, 2014; Mills et al., 2017; Janssen et al., 2018). A review of titles and abstracts narrowed this down to 36 papers deemed to be of potential relevance. A full-text review of these 36 papers was then undertaken, which resulted in ten papers meeting all of the eligibility criteria (Appendix 1: Reasons for excluding the full-text-reviewed articles). The PRISMA flow diagram for the article selection process is shown in Figure 1.

### Characteristics of Included Studies

#### Published Year

All studies were published in the last decade (2014-2019).

#### Location of Research

Six studies were conducted in Europe (Beshai et al., 2016; Sansó et al., 2017, 2019; Delaney, 2018; Maratos et al., 2019; Smith et al., 2019), two studies were conducted in North America (Rao and Kemper, 2017; Scarlet et al., 2017), one study was conducted in Oceania (Brooker et al., 2013; Pidgeon et al., 2014) and another study was conducted in Asia (Suyi et al., 2017).

#### Design

One study used randomized controlled trial designs (Pidgeon et al., 2014), two used non-equivalent groups design (Beshai et al., 2016; Sansó et al., 2019), and seven studies used a within-subject pre-post design (Rao and Kemper, 2017; Sansó et al., 2017; Scarlet et al., 2017; Suyi et al., 2017; Delaney, 2018; Maratos et al., 2019; Smith et al., 2019).

#### Participant Demographics

All studies (that reported gender balance) recruited more females than males, including one study that recruited women only (Delaney, 2018). The sample size ranged from 13 (Delaney, 2018) to 153 participants (Rao and Kemper, 2017), totaling 512 participants overall. The most common age range of participants across all studies was 40-50 years.

#### Occupational Field

Participants in eight studies worked in healthcare fields including doctors, nurses, social workers, human service professionals, and disability support workers (Pidgeon et al., 2014; Rao and Kemper, 2017; Sansó et al., 2017, 2019; Scarlet et al., 2017; Suyi et al., 2017; Delaney, 2018; Smith et al., 2019). Participants in two studies worked in education (Beshai et al., 2016; Maratos et al., 2019).

#### Scale

Four studies used the original version of the Self-Compassion Scale (SCS; Neff, 2003b; Pidgeon et al., 2014; Sansó et al., 2017, 2019; Delaney, 2018) and one study used two subscales of the SCS (self-kindness and self-judgement; Beshai et al., 2016). The remaining four studies used the Self-Compassion Scale-Short Form (SCS-SF; Raes et al., 2011; Rao and Kemper, 2017; Scarlet et al., 2017; Suyi et al., 2017; Maratos et al., 2019), and one study used the Five-Facets Mindfulness Questionnaire (FFMQ; Cebolla et al., 2012) to measure the effects of self-compassion training (Smith et al., 2019).

### Interventions

Six studies used interventions with a central self-compassion component: Compassionate Mind Training (lectures on brain, breathing practice, imagery practice; Maratos et al., 2019), Compassion Cultivation Training (lectures on the mind, meditation practice focusing on self-compassion and loving-kindness; Scarlet et al., 2017; Sansó et al., 2019), online meditation training on self-compassion (psycho-education, meditation practice, and discussion; Rao and Kemper, 2017), a well-being program focusing on self-compassion (workshops about yoga, communication, and others; Smith et al., 2019), and Cultivating Emotional Balance program (theoretical presentations, discussions, and guided practice exercises of meditation and emotion regulation; Sansó et al., 2017). Four studies used a combination of mindfulness and self-compassion training (Pidgeon et al., 2014; Beshai et al., 2016; Suyi et al., 2017; Delaney, 2018). The total duration of interventions ranged from 1 h (Rao and Kemper, 2017) to 60 h (Sansó et al., 2019), and five studies assigned daily practice as homework (Beshai et al., 2016; Sansó et al., 2017; Scarlet et al., 2017; Suyi et al., 2017; Delaney, 2018), ranging from 20 min (Scarlet et al., 2017) to 30 min (Suyi et al., 2017).

Three studies used one follow-up assessment (Sansó et al., 2017; Scarlet et al., 2017; Suyi et al., 2017) and one study used two follow-up assessments (1 and 4 months after intervention; Pidgeon et al., 2014). Only one study used two pre-intervention assessments (2-month and 1-week before intervention; Maratos et al., 2019). A 1-month follow-up was the shortest follow-up period (Scarlet et al., 2017), and the longest was 6 months (Sansó et al., 2017). In addition to self-compassion, 17 psychological outcomes were measured across all studies, of which common outcomes included mindfulness, stress and burnout. Table 3 summarizes the intervention characteristics, outcomes and follow-up assessment periods.

**Table 3:** Intervention, psychological outcomes, and follow-up in the 10 included studies.

**No**.	**References**	**Intervention**		Other outcomes than self-compassion^*^							**Follow-up**
		**Length of intervention in total (hr)**	**Daily homework (min)**	**Mindfulness**	**Stress**	**Burnout**	**Quality of life**	**Mental well-being**	**Resilience**	**Others**	
1	Maratos et al. (2019)	15 (6 × 2.5-h sessions)	0		✓	✓				Anxiety, Depression, Self-criticism/-reassurance	None (pre-assessments at 2-month and 1-week)
2	Sansó et al. (2019)	3 (3 × 1-h sessions)	0	✓			✓			Interpersonal reactivity	None
3	Smith et al. (2019)	Not reported	0					✓			None
4	Delaney (2018)	20 (8 × 2.5-h sessions)	Assigned but details unreported	✓			✓		✓		None
5	Rao and Kemper (2017)	1 (1-h session)	0							Confidence in caregiving	None
6	Sansó et al. (2017)	42 (10 × 4-h sessions + 1 × 2-h session)	Assigned but details unreported	✓						Self-care	6 months
7	Scarlet et al. (2017)	16 (8 × 2-h sessions)	20	✓		✓				Fear of compassion, job satisfaction, interpersonal conflict	1 month
8	Suyi et al. (2017)	12 (6 × 2-h sessions)	30	✓	✓	✓				Compassion for others	3 months
9	Beshai et al. (2016)	11.25 (9 × 1.25-h sessions)	25	✓	✓			✓			None
10	Pidgeon et al. (2014)	13 (2.5 h + 1 h + 2.5 h + 2.5 h + 1 h +1 h + 2.5 h)	0	✓					✓		1 and 4 months

* *All studies assessed self-compassion (see Table 2)*.

### Risk of Bias

The risk of bias in the nine non-randomized studies was mostly deemed to be medium, indicated by a rating of 3-4 stars (medium: Rao and Kemper, 2017; Sansó et al., 2017; Scarlet et al., 2017; Suyi et al., 2017; Delaney, 2018; Maratos et al., 2019; Smith et al., 2019). Only two non-randomized studies received a low bias rating of 6-7 stars (low: Beshai et al., 2016; Sansó et al., 2019). All of the non-randomized studies assessed psychological outcomes before and after the intervention. Two studies (Beshai et al., 2016; Delaney, 2018) addressed the representativeness of the cohort by recruiting a sample that is comparable with the general population of the target workers, and one study (Beshai et al., 2016) addressed adequacy of follow-up by providing reasons for dropout at follow-up (see Table 4).

**Table 4:** Assessment of quality of studies (non-randomized trials; nine studies).

**Assessment of risk of bias for pre-post studies (The Newcastle-Ottawa scale)**									
	**Selection**				**Comparability**	**Outcome**			**Number of stars (0-9)**
**References**	**Representativeness of exposed cohort**	**Selection of non-exposed cohort**	**Ascertainment of exposure**	**Demonstrate outcome assessed before intervention**	Comparability of cohorts on basis of design (^*^) or analysis (^*^)	**Assessment of outcome**	**Follow-up long enough**	**Adequacy of follow-up**	
Maratos et al. (2019)			*	*		*			3
Sansó et al. (2019)		*	*	*	**	*			6
Smith et al. (2019)			*	*		*			3
Delaney (2018)	*		*	*		*			4
Rao and Kemper (2017)			*	*		*			3
Sansó et al. (2017)			*	*		*	*		4
Scarlet et al. (2017)			*	*		*	*		4
Suyi et al. (2017)			*	*		*	*		4
Beshai et al. (2016)	*	*	*	*	**			*	7

* *The study addressed the assessment item*.

The risk of bias for the only RCT in the included studies (Pidgeon et al., 2014) was deemed medium (6/12) (Table 5). Because this study employed a passive control group, three assessment items, blinding for assessors, administration, and participants were scored “NA” (Not Applicable)'. The number of participants randomly allocated to both groups (intervention and control), baseline comparability, inclusion criteria and reasons for withdrawal were reported. However, it was not reported as to whether allocation of treatment was concealed, the blinding procedure was successful, and reported outcomes were the only outcomes assessed. Furthermore, <80% of the original participants completed the final assessment, and an intention-to-treat analysis was not conducted.

**Table 5:** Assessment of quality of studies based on mental health outcome (randomized controlled trials).

**Quality assessment table of randomized controlled trials (Brown et al.**, **2013****)**																
	**Randomization**			**Baseline comparability**		**Inclusion criteria specified**	**Co-interventions identified**	**Blinding**				**Withdrawals**		**Intention to treat**	**Other outcomes**	**Score**
**Reference**	**Truly random**	**Allocation concealment**	**Number stated**	**Presented**	**Achieved**			**Assessors**	**Administration**	**Participants**	**Procedure assessed**	**>80% in final analysis**	**Reasons stated**			
Pidgeon et al. (2014)	*		*	*	*	*		NA	NA	NA			*			6/12

^*^The study addressed the assessment item.

## Discussion

This PRISMA-based systematic review appraised the quality of evidence for eligible studies evaluating the effects of self-compassion training on work-related well-being, addressing (i) how effective self-compassion is for work-related well-being outcomes, and (ii) what quantity and quality of evidence has been reported. Ten intervention studies (nine pre-post within-subject studies and one RCT), comprising a total of 512 participants, met all of the eligibility criteria for in-depth review and assessment. Self-compassion was shown to improve in all studies, including those deemed to be more methodologically robust (e.g., Beshai et al., 2016; Sansó et al., 2019). Improvements were also reported across other outcomes, such as mindfulness, stress, burnout, quality of life, well-being, and resilience. Thus, findings from this systematic review indicate that PP 2.0 interventions based on self-compassion have applications for improving workplace mental health.

The total length of the interventions varied from 1 to 60 h, however high-quality studies (Beshai et al., 2016; Sansó et al., 2019) employed at least 11.25 h of intervention, delivered over an 8-week period (Beshai et al., 2016). While one study reported that a 1-h online session of self-compassion training led to improvements in self-compassion (Rao and Kemper, 2017), results should be interpreted with caution as a follow-up assessment was not conducted, and several confounding factors were identified. Thus, based on the ten studies included in this systematic review, the most robust evidence exists for self-compassion interventions of longer duration (i.e., >11 h) spread across several sessions (i.e., with self-practice elements in between).

The majority of studies included in this review were conducted in Western countries. However, self-compassion can be perceived differently between cultures (Montero-Marin et al., 2018). For example, the positive aspects of self-compassion are deemed to assume greater importance in cultures that assign virtue to future-focused strategies of perseverance and thrift (Hoofstede et al., 2010). This cultural characteristic is thought to arise as a result of positive attitudes toward challenges in learning, whereby self-compassion is deemed to temper the need for instant gratification or short-term reward (Wagner et al., 2017). Thus, in line with the use of Eastern philosophical principles in PP 2.0, evaluating the effects of self-compassion training for workers in countries that appear to value a longer-term reward orientation, such as China, Taiwan, and Hong Kong, could advance understating into the cross-cultural applications of self-compassion training. Similarly, given that all participants in the ten included studies were workers in a caring profession or educational profession (i.e., key workers during the COVID-19 pandemic), future research should explore how different industry cultures influence the effects of self-compassion training.

In the studies included in this systematic review, self-compassion was measured using the SCS (Neff, 2003b) and SCS-SF (Raes et al., 2011). While these measures are commonly used in self-compassion research (López et al., 2015), the appropriateness of SCS and SCS-SF as a measurement for self-compassion has been questioned (López et al., 2015; Kotera and Sheffield, 2020). For example, the negative subscales of SCS have been shown to be more strongly related to depression, stress and rumination than the positive ones (López et al., 2015), and the originally proposed factor structure of the SCS-SF (Raes et al., 2011) was not replicated in a study of UK university students (Kotera and Sheffield, 2020). Future studies in this area may thus benefit from assessing self-compassion using scales such as the Relational Compassion Scale (Hacker, 2008) or Compassionate Engagement and Action Scales (Gilbert et al., 2017). Alternatively, considering the aforementioned Eastern influence of PP 2.0 (Wong, 2011a), scales that were originally developed in Eastern languages that are completed by native language speakers (i.e., instead of translating English language scales) may be able to better capture some of the subtle nuances of self-compassion for given cultural groups. For example, the Omoiyari (roughly translated as compassion in Japanese) Scale (Uchida and Kitayama, 2001) may capture more accurate responses regarding self-compassion for native Japanese-speaking participants.

The quality of the included studies overall was medium: eight studies were assessed to bear a medium risk of bias (Rao and Kemper, 2017; Sansó et al., 2017; Scarlet et al., 2017; Suyi et al., 2017; Delaney, 2018; Maratos et al., 2019; Smith et al., 2019), and two studies were assessed to bear a low risk of bias (Beshai et al., 2016; Sansó et al., 2019). Among the non-randomized studies, most studies did not address (i) representativeness of the exposed cohort, (ii) selection of the non-exposed cohort, (iii) comparability of cohorts, and (iv) follow-up adequacy. The only RCT included in this systematic review (Pidgeon et al., 2014) was assessed to bear a medium risk of bias. In this instance, concealment of allocation, assessment of the blinding procedure, and whether there were co-interventions and/or other assessed outcomes, were not reported. Moreover, the intervention completion rate fell below 80% and an intention-to-treat analysis was not conducted. More methodologically robust randomized controlled trials of self-compassion studies in worker populations are thus needed to address these issues, which has been identified as a need underlying research of this nature within contemporary organizational psychology (Gubbins and Rousseau, 2015).

While this systematic review offers helpful insights into the applications of self-compassion in a workplace context, several limitations should be noted. Unpublished studies or studies not published in English language were not considered, meaning that some relevant evidence pertaining to self-compassion at work may have been overlooked. Furthermore, this review only included interventions that explicitly described the target intervention as incorporating a notable self-compassion component, and may thus have excluded studies of interventions where self-compassion is taught more implicitly. For example, positive reframing to provide compassionate views on one's own and others' weaknesses has been reported as an effective workplace technique by Japanese senior managers (Kotera and Van Gordon, 2019). While such techniques are not framed as a self-compassion interventions *per se*, future systematic reviews could employ a broader definition of self-compassion.

The ten studies included in this systematic review indicate that self-compassion training can be effective for improving work-related well-being outcomes. However, research in this area is relatively new and has largely focused on key workers who work in caring and education professions, primarily in Western countries. Evaluating the applications of self-compassion in other work contexts is important because studies have shown that individuals working in, for example, construction, are not always comfortable with the self-compassion construct and relate it to more as self-pity (Kotera et al., 2019b). Similarly, there appears to be a different perception of self-compassion in certain Asian countries, which should be explored further as part of future research. This is particularly essential as PP 2.0 is influenced by the Eastern philosophy. Efforts should also be made to improve the methodological rigor of self-compassion intervention studies, which in the current systematic review were mostly of a moderate study quality. In particular, more randomized controlled trials with larger sample sizes and an active control condition are required to draw better comparisons between the benefits to work-related well-being of self-compassion training based on the concept of PP 2.0, as well as related techniques such as mindfulness.

## Data Availability Statement

The original contributions presented in the study are included in the article/Supplementary Material, further inquiries can be directed to the corresponding author/s.

## Author Contributions

YK: conceived, designed this systematic review, collected, and organized the data. YK and WVG: performed the analysis and wrote the paper.

## Conflict of Interest

The authors declare that the research was conducted in the absence of any commercial or financial relationships that could be construed as a potential conflict of interest.
