# Supplementary material for: Effects of Self-Compassion Training on Work-Related Well-Being: A Systematic Review
Source: Front Psychol. 2021 Apr 23;12:630798. doi: 10.3389/fpsyg.2021.630798 (PMC8102699; doi:10.3389/fpsyg.2021.630798)
Supplement: Supplementary file 1 [file Table_1.docx]

Supplementary Material

Effects of Self-Compassion Training on Work-Related Wellbeing: A Systematic Review

*Appendix 1. Reasons for excluding the full‐text‐reviewed articles*

| **Author(s), year** | **Reason** |
| --- | --- |
| Pérula-de Torres et al., 2019 | Not empirical |
| Held and Owens, 2015 | Not workers |
| Newsome et al., 2012 | Not workers |
| Kinman and Grant, 2017 | Not self-compassion training |
| Strauss et al., 2018 | Not empirical |
| Rodrigues et al., 2018 | Not self-compassion training |
| Adimando, 2018 | Not self-compassion training |
| Smith et al., 2019 | Not self-compassion training |
| Orellana-Rios et al., 2017 | Not measuring self-compassion |
| Trent et al., 2019 | Not self-compassion training |
| Luthar et al., 2017 | Not self-compassion training |
| Ho et al., 2019 | Not empirical |
| Whitesman and Mash, 2016 | Not empirical |
| Wasner et al., 2005 | Not measuring self-compassion |
| Whitebird et al., 2018 | Not workers |
| Lopes et al., 2019 | Not self-compassion training |
| Gasper et al., 2018 | Not self-compassion training |
| Arredondo et al., 2017 | Not self-compassion training |
| Raab et al., 2017 | Not self-compassion training |
| dos Santos et al., 2016 | Not self-compassion training |
| Gauthier et al., 2015 | Not self-compassion training |
| Brooker et al., 2013 | Not self-compassion training |
| Frank, 2013 | Not self-compassion training |
| Roeser et al., 2013 | Not self-compassion training |
| Shapiro et al., 2005 | Not self-compassion training |
| Simola, 2019 | Not workers |

References

Adimando, A. (2018). Preventing and Alleviating Compassion Fatigue Through Self-Care: An Educational Workshop for Nurses. *Journal of Holistic Nursing*, *36*(4), 304–317. https://doi.org/10.1177/0898010117721581

Arredondo, M., Sabaté, M., Valveny, N., Langa, M., Dosantos, R., Moreno, J., & Botella, L. (2017). A mindfulness training program based on brief practices (M-PBI) to reduce stress in the workplace: a randomised controlled pilot study. *International Journal of Occupational and Environmental Health*, *23*(1), 40–51. https://doi.org/10.1080/10773525.2017.1386607

Brooker, J., Julian, J., Webber, L., Chan, J., Shawyer, F., & Meadows, G. (2013). Evaluation of an Occupational Mindfulness Program for Staff Employed in the Disability Sector in Australia. *Mindfulness*, *4*(2), 122–136. https://doi.org/10.1007/s12671-012-0112-7

Dos Santos, T. M., Kozasa, E. H., Carmagnani, I. S., Tanaka, L. H., Lacerda, S. S., & Nogueira-Martins, L. A. (2016). Positive effects of a stress reduction program based on mindfulness meditation in brazilian nursing professionals: Qualitative and quantitative evaluation. *Explore: The Journal of Science and Healing*, *12*(2), 90–99. https://doi.org/10.1016/j.explore.2015.12.005

Frank, G. (2013). A system-based approach to home modifications for the catastrophically injured. *Journal of Nurse Life Care Planning*, *13*(4), 138–146.

Gasper, A. M., Magdic, K., Ren, D., & Fennimore, L. (2018). Development of a home health-based palliative care program for patients with heart failure. *Home Healthcare Now*, *36*(2), 84–92. https://doi.org/10.1097/NHH.0000000000000634

Gauthier, T., Meyer, R. M. L., Grefe Phd, D., & Gold Phd, J. I. (2015). *An On-the-Job Mindfulness-based Intervention For Pediatric ICU Nurses: A Pilot*. https://doi.org/10.1016/j.pedn.2014.10.005

Held, P., & Owens, G. P. (2015). Effects of Self-Compassion Workbook Training on Trauma-Related Guilt in a Sample of Homeless Veterans: A Pilot Study. *Journal of Clinical Psychology*, *71*(6), 513–526. https://doi.org/10.1002/jclp.22170

Ho, A. H. Y., Tan-Ho, G., Ngo, T. A., Ong, G., Chong, P. H., Dignadice, D., & Potash, J. (2019). A novel mindful-compassion art therapy (MCAT) for reducing burnout and promoting resilience for end-of-life care professionals: A waitlist RCT protocol. *Trials*, *20*(1), 406. https://doi.org/10.1186/s13063-019-3533-y

Kinman, G., & Grant, L. (2017). Building Resilience in Early-Career Social Workers: Evaluating a Multi-Moda...: LIBRARY PLUS - for books, articles and more. *British Journal of Social Work*, *47*(7), 1979–1998.

Lopes, S. A., Vannucchi, B. P., Demarzo, M., Cunha, Â. G. J., & Nunes, M. do P. T. (2019). Effectiveness of a Mindfulness-Based Intervention in the Management of Musculoskeletal Pain in Nursing Workers. *Pain Management Nursing*, *20*(1), 32–38. https://doi.org/10.1016/j.pmn.2018.02.065

Luthar, S. S., Curlee, A., Tye, S. J., Engelman, J. C., & Stonnington, C. M. (2017). Fostering Resilience among Mothers under Stress: “Authentic Connections Groups” for Medical Professionals. *Women’s Health Issues : Official Publication of the Jacobs Institute of Women’s Health*, *27*(3), 382–390. https://doi.org/10.1016/j.whi.2017.02.007

Newsome, S., Waldo, M., & Gruszka, C. (2012). Mindfulness Group Work: Preventing Stress and Increasing Self-Compassion Among Helping Professionals in Training. *Journal for Specialists in Group Work*, *37*(4), 297–311. https://doi.org/10.1080/01933922.2012.690832

Orellana-Rios, C. L., Radbruch, L., Kern, M., Regel, Y. U., Anton, A., Sinclair, S., & Schmidt, S. (2017). Mindfulness and compassion-oriented practices at work reduce distress and enhance self-care of palliative care teams: A mixed-method evaluation of an “on the job” program. *BMC Palliative Care*, *17*(1). https://doi.org/10.1186/s12904-017-0219-7

Pérula-De Torres, L. A., Atalaya, J. C. V. M., García-Campayo, J., Roldán-Villalobos, A., Magallón-Botaya, R., Bartolomé-Moreno, C., Moreno-Martos, H., Melús-Palazón, E., Liétor-Villajos, N., Valverde-Bolívar, F. J., Hachem-Salas, N., Rodríguez, L. A., Navarro-Gil, M., Epstein, R., Cabezón-Crespo, A., Moreno, C. M. V., Vega, R. A., Salguero, A. F., López, I. A., … Camacho, A. S. (2019). Controlled clinical trial comparing the effectiveness of a mindfulness and self-compassion 4-session programme versus an 8-session programme to reduce work stress and burnout in family and community medicine physicians and nurses: MINDUUDD study protocol. *BMC Family Practice*, *20*(1). https://doi.org/10.1186/s12875-019-0913-z

Raab, K. (2014). Mindfulness, Self-Compassion, and Empathy Among Health Care Professionals: A Review of the Literature. *Journal of Health Care Chaplaincy*, *20*(3), 95–108. https://doi.org/10.1080/08854726.2014.913876

Rodrigues, N., Cohen, L., McQuarrie, S., & Reed-Knight, B. (2018). *Burnout in nurses working with youth with chronic pain: A pilot intervention.* *43*(4), 382–391.

Roeser, R. W., Schonert-Reichl, K. A., Jha, A., Cullen, M., Wallace, L., Wilensky, R., Oberle, E., Thomson, K., Taylor, C., & Harrison, J. (2013). Mindfulness training and reductions in teacher stress and burnout: Results from two randomized, waitlist-control field trials. *Journal of Educational Psychology*, *105*(3), 787–804. https://doi.org/10.1037/a0032093

Shapiro, S. L., Astin, J. A., Bishop, S. R., & Cordova, M. (2005). Mindfulness-Based Stress Reduction for Health Care Professionals: Results From a Randomized Trial. *International Journal of Stress Management Publishing Foundation*, *12*(2), 164–176. https://doi.org/10.1037/1072-5245.12.2.164

Simola, S. K. (2019). Educating business students to manage for mental health. *Journal of Mental Health Training, Education and Practice*, *14*(5), 315–326. https://doi.org/10.1108/JMHTEP-01-2019-0005

Smith, E., Willis, E., & McDonagh, J. (2019). P33 Adolescent and young adult health training needs of healthcare professionals including rheumatology. *Rheumatology*, *58*(Supplement_4). https://doi.org/10.1093/rheumatology/kez416

Strauss, C., Gu, J., Pitman, N., Chapman, C., Kuyken, W., & Whittington, A. (2018). Evaluation of mindfulness-based cognitive therapy for life and a cognitive behavioural therapy stress-management workshop to improve healthcare staff stress: Study protocol for two randomised controlled trials. *Trials*, *19*(1), 209. https://doi.org/10.1186/s13063-018-2547-1

Trent, N. L., Borden, S., Miraglia, M., Pasalis, E., Dusek, J. A., & Khalsa, S. B. S. (2019). Improvements in Psychological and Occupational Well-being Following a Brief Yoga-Based Program for Education Professionals. *Global Advances in Health and Medicine*, *8*, 216495611985685. https://doi.org/10.1177/2164956119856856

Wasner, M., Longaker, C., Fegg, M. J., & Borasio, G. D. (2005). Effects of spiritual care training for palliative care professionals. *Palliative Medicine*, *19*(2), 99–104. https://doi.org/10.1191/0269216305pm995oa

Whitebird, R. R., Kreitzer, M. J., Vazquez-Benitez, G., & Enstad, C. J. (2018). Reducing diabetes distress and improving self-management with mindfulness. *Social Work in Health Care*, *57*(1), 48–65. https://doi.org/10.1080/00981389.2017.1388898

Whitesman, S., & Mash, R. (2016). Examining the effects of a mindfulness-based distance learning professional training module on personal and professional functioning: a qualitative study. *BMC Medical Education*, *16*(1), 1–8. https://doi.org/10.1186/s12909-016-0810-2
